# Femtosecond Laser Texturing of Wood Coatings with Bio-Based Epoxy and Wax Additives for Enhanced Hydrophobicity

**DOI:** 10.3390/mi17060759

**Published:** 2026-06-22

**Authors:** Pieter Samyn, Patrick Cosemans, Olivier Malek

**Affiliations:** 1 Department of Innovations in Circular Economy and Renewable Materials, SIRRIS, 3001 Leuven, Belgium; patrick.cosemans@sirris.be; 2 Department of Manufacturing Systems and Technologies, SIRRIS, 3600 Genk, Belgium; olivier.malek@sirris.be

**Keywords:** laser texturing, femtosecond, epoxy, wax, coating, hydrophobicity

## Abstract

Femtosecond laser surface texturing offers a promising route to tailor the functionality of bio-based wood coatings, yet the interplay between coating composition and laser processing remains poorly understood. In this study, bio-based epoxy coatings with eventual micronized wax additives were textured using a femtosecond laser to investigate the effects of laser processing parameters on pattern formation and resulting hydrophobicity. The epoxy coatings containing PE, PE/PTFE, HDPE, and rice bran waxes at 1, 5, and 7 wt.-% were characterized in terms of morphology, roughness, wettability, and chemical stability, followed by systematic variation of pulse repetition rate and laser power. The results reveal that the ablation threshold strongly depends on intrinsic coating properties. Ablation resistance increases with surface roughness and wax melting enthalpy, reflecting the role of phase transition energy in laser–matter interaction. The wax-filled coatings exhibit a transition from melting-dominated behavior at low energy input to ablation-dominated behavior at a higher energy. Laser texturing enhances hydrophobicity in parallel with theoretical values calculated from the Cassie–Baxter wetting model, with the highest hydrophobicity achieved for coatings combining intrinsic hydrophobicity and stable pattern formation. Chemical analysis confirms limited degradation of the epoxy matrix without significant carbonization, while wax additives provide partial thermal shielding. Overall, this work demonstrates clear options for tailoring surface morphology and wettability of hydrophobic polymer coatings through controlled femtosecond laser processing.

## 1. Introduction

The protective wood coatings play a crucial role in enhancing the durability, aesthetics, and functional performance of wood-based materials, particularly in applications exposed to outdoor or humid environments [1]. Among the desired functionalities, hydrophobicity is of primary importance, as water uptake is a key driver of degradation processes such as swelling, biological attack, and coating delamination. Conventional approaches to impart water repellence rely on modifying the chemical composition of coatings through the incorporation of low-surface-energy components, traditionally including fluorinated additives and copolymers [2]. The combination of TiO_2_ nanoparticles and perfluorinated substances (PFASs) is frequently reported as a solution for superhydrophobic wood coatings [3]. However, the more sustainable approaches avoiding toxic and persistent chemicals should introduce environmentally benign substances or bio-based chemicals based on natural waxes [4], bark components [5], lignin [6], siloxane–nano–lignin [7], and cellulose nanocrystals [8] that are used as binders or additives. In addition to the use of synthetic polymers, several studies reported on the construction of superhydrophobic coatings from biomass materials such as lignin, cellulose, chitosan, and starch [9]. In addition, the control of surface morphology becomes increasingly recognized as a critical parameter in determining the wetting behavior [10]. Some appealing approaches for biomimetic superhydrophobic wood coatings with extended functionality were explored [11], including graft copolymerization, chemical vapor deposition, hydrothermal synthesis, sol–gel methods, template methods, dip coating, and spraying methods. At present, methods such as the sol–gel method, the etching method, graft copolymerization, and the layer-by-layer self-assembly method can be used to prepare superhydrophobic surfaces [12]. The combination of chemical moieties based on zeolitic imidazole frameworks (ZIF-8 nanocrystals), paraffin, and hexadecyltrimethoxysilane at a specific ratio demonstrated the ability to provide a surface microtexture required for superhydrophobicity [13]. However, the frequent use of nanoparticles (TiO_2_, ZnO, Ag, silica) in superhydrophobic coatings may introduce specific environmental, human health, and performance risks due to potential leaching and accumulation in soil and water [14]. In line with industrial practice, chemical modification should be limited, and coating formulation with known ingredients (e.g., mixing of binder + additives) is preferred in combination with scalable post-processing. Therefore, future advances should be made in the design of superhydrophobic coatings by combining environmentally friendly components and processing technologies while avoiding nanotechnology [15].

In recent years, surface texturing has emerged as a powerful tool to tailor interfacial properties of polymer composites [16], polymeric coatings [17,18,19], and hydrophobic cellulose coatings [20] by creating controlled surface topographies. The combination of surface chemistry and hierarchical roughness can induce a transition from Wenzel to Cassie–Baxter wetting regimes, significantly increasing apparent contact angles [21]. As a result, the effect of surface texturing on wettability can be modeled on either isotropic or anisotropic surfaces [22]. Among the various texturing techniques, the ultrafast laser processing—particularly femtosecond laser texturing—has gained considerable attention due to its ability to generate well-defined micro- and nanoscale structures with minimal thermal damage [23]. In contrast to nanosecond or continuous-wave lasers, femtosecond pulses enable highly localized energy deposition through nonlinear absorption mechanisms, allowing precise material removal (“cold ablation”) and controlled surface structuring [24]. The latter has been well-documented on hard ceramic and metal surfaces [25,26]. Despite the growing application of femtosecond laser texturing and its suitability for soft materials, its application in polymeric coatings remains limited [27]. However, the understanding of laser–matter interaction in complex systems becomes prevalent in heterogeneous coating systems [28,29]. In particular, the interplay between intrinsic material properties—such as thermal transitions, rheological behavior, and phase composition—and laser processing parameters has to be interpreted for optimization of the laser processing in polymers [30]. This is especially relevant for hybrid coating systems where thermosetting matrices, such as epoxy resins, are combined with dispersed thermoplastic or crystalline additives, like waxes, that are selected to promote hydrophobicity [31]. Micronized wax particles are commonly used in coatings to modify surface properties through migration, phase separation, and micro-roughness formation during curing [32]. However, their presence introduces additional complexity under ultrafast laser irradiation, as low melting temperatures and distinct phase transition energetics may promote melting, flow, or vaporization processes that compete with ablation.

In this context, the present study investigates the femtosecond laser surface texturing of bio-based epoxy coatings with micronized wax additives, with a particular focus on the role of laser pulse rate and power setting on pattern stability and hydrophobicity. The main objective is to elucidate the governing mechanisms that determine the effectiveness of femtosecond laser texturing in enhancing the hydrophobicity of the coatings. Particular attention is given to the transition between melting-dominated and ablation-dominated regimes, the influence of wax phase transitions on ablation thresholds, and the resulting wetting behavior in relation to theoretical models. The findings aim to provide fundamental insights and practical guidelines for the design of advanced, sustainable wood coatings with tunable surface functionalities using scalable laser-based technologies.

## 2. Materials and Methods

### 2.1. Materials

The wood substrates consist of 10 × 10 cm^2^ samples of hardwood beech with a thickness of 5 mm and a planed top surface, as obtained from a local shop (Martens Hout, Leuven, Belgium). After milling, the wood samples were dried overnight in a hot circulating air oven at 60 °C to control moisture content before the coatings were applied.

A 100% bio-based epoxy grade was supplied by Orineo BV (Kortenberg, Belgium) and consists of a proprietary component A (epoxidized vegetable oil) and component B (bio-based acid mixture) that were mixed according to the supplier’s recommended stoichiometric ratio (A:B = 100:45 by weight) to achieve optimal crosslinking. The system forms a thermoset network through ring-opening reactions of the epoxide groups with the acid functionalities. Although the exact formulation is proprietary, its key characteristics include an optimized low viscosity suitable for coating applications and curing temperatures in the range of 60 to 90 °C.

The different types of fossil-based and bio-based micronized wax powders were supplied by BYK (Wesel, Germany), including PE wax, PE/PTFE wax (75/25 ratio), HDPE wax, and a proprietary grade of rice bran (RB) wax. The selected micronized powder grades have average mean sizes (d_50_) of 5 to 7 µm.

### 2.2. Sample Preparation

The bio-based epoxy wood coatings were formulated by mixing component A and component B together with 1, 5, and 7 wt.-% of the respective micronized wax particles. The reference epoxy coating without wax additives was obtained by similar mixing and processing of the epoxy ingredients. A typical batch contained 20 g of material that was applied onto the wood substrates by blade coating into a wet coating thickness of about 500 µm, corresponding to a 485 to 490 µm dry coating thickness. The thermal crosslinking of the bio-based epoxy coatings was done for 1 h at 60 °C, followed by 3 h at 90 °C.

### 2.3. Laser Texturing

The small areas of 1 × 1 cm^2^ on the coated wood samples were patterned through femtosecond laser surface texturing on a LS5 workstation (Lasea, Liège, Belgium) with samples mounted on a X, Y, Z stage controller. The laser source is a Yb-doped near-infrared laser with 1030 nm wavelength (Stasuma HP, Amplitude Systems, Pessac, France) and a maximum 10 W laser output power, operating under atmospheric conditions in the femtosecond range (250 fs pulses). The laser beam has a Gaussian profile and is focused to a single spot with a diameter of 17 μm at the sample plane using an f-theta lens with a focal length of 100 mm. The variable laser parameters were applied in order to investigate the sensitivity of the coating to the laser patterning process, i.e., after preliminary determination of the applicable operational ranges (see Supplementary Information S1). The laser power setting was varied in discrete steps between 50 and 60% at a constant scanning speed of 4000 mm/s and pulse rate (or so-called pulse repetition rate) of 250 or 500 kHz. The corresponding laser fluence values under given conditions within operational ranges are summarized in Table 1. Within the scope of the designed feasibility study to investigate the effect of pulse repetition rate and laser power setting on patterning effects of the coatings, a fixed hatch pitch distance of 35 µm was selected to ensure controlled and reproducible surface structuring while avoiding inter-track interference. This spacing relative to the 17 µm laser spot size and Gaussian beam profile provides moderate overlap that maintains continuous pattern coverage without excessive thermal accumulation. By keeping the hatch pitch constant, the study isolates the effects of laser power and pulse repetition rate on pattern formation, enabling direct interpretation of melting- versus ablation-dominated regimes. Furthermore, the fixed periodicity ensures consistency with the geometrical parameters used in Cassie–Baxter modeling.

### 2.4. Characterization Techniques

The microscopic evaluations were made through laser interference microscopy on the VK-X3000 microscope (Keyence, Mechelen, Belgium). The analysis allows for a visual inspection of the surfaces (laser image, optical image) and the topographical data analysis (height image, 3D topography). The average surface roughness Sa was determined on a standardized sampling area (700 × 500 µm^2^) at an objective lens magnification of 20× (688 nm/pixel, Z calibration 0.1 nm/digit, Z measurement distance 67.7 µm), and a broader view on surface homogeneity was obtained on a sampling area (2700 × 2000 µm^2^) with an objective lens magnification of 5× (2690 nm/pixel, Z calibration 0.1 nm/digit, Z measurement distance 730 µm). All images were systematically pre-processed through secant curved surface tilting (automatically) in VK Analyzer Software (version 2006, 3.9.50.0, Keyence, Mechelen, Belgium).

The surface hydrophobicity was evaluated through static contact angles with deionized water on a OCA50 goniometer (Dataphysics Instruments GmbH, Filderstadt, Germany), using a 3 µL droplet volume that was deposited and geometrically fitted with a Laplace–Young algorithm, following ISO 19403-2 [33]. Measurements were repeated 3 times per patterned surface area (1 × 1 cm^2^), with an average standard variation of ±3°.

Fourier-transform infrared (FTIR) spectra were acquired using attenuated total reflection (ATR) with a diamond crystal and a HeNe laser on a Nicolet iS10 spectrometer (Thermo Fisher Scientific, Breda, The Netherlands). Spectra were collected over the wavenumber range of 4000–500 cm^−1^ at a resolution of 4 cm^−1^, with each spectrum averaged over 32 scans.

The mechanical properties of native coatings (before laser texturing), including microhardness, were determined with a Shore D durometer with a hardened steel tip with a 30 ± 0.5° conical point and a 0.100 ± 0.012 mm tip radius following ASTM D2240 [34]. The specular gloss measurements were performed with a micro-TRI-gloss meter (BYK-Gardner Instruments, Geretsried, Germany) under 60° following ISO 2813 [35].

## 3. Results and Discussion

### 3.1. Identification of Intrinsic Coating Properties

**Figure 1:**
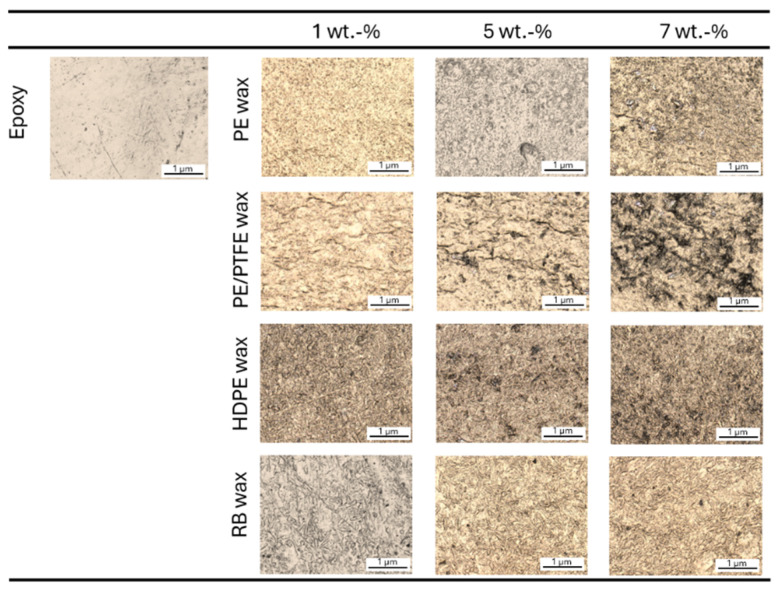
Detailed optical microscopy of native epoxy coatings with different concentrations (1, 5, 7 wt.-%) of micronized wax additives.

**Figure 2:**
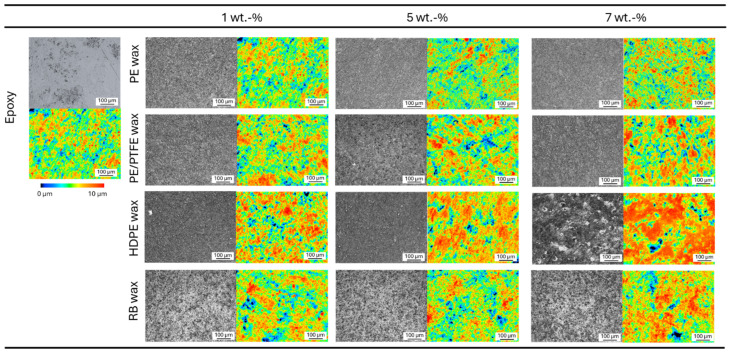
Laser interference microscopy and topographical analysis of native epoxy coatings with different concentrations (1, 5, 7 wt.-%) of micronized wax additives. The same color scale bar (z-scale) applies to all topographical images.

The different formulations of epoxy coatings with 1, 5, and 7 wt.-% of micronized wax additives were first characterized by initial morphology and intrinsic properties in order to potentially relate further to the efficiency of the laser texturing process. As shown in Figure 1, the detailed optical micrographs (150× objective lens) of the coatings indicate a smooth surface for the pure epoxy coating, while irregular features are introduced at the surface of wax-filled epoxy coatings. The latter are quantifiable by topographical analysis of the coatings at a larger scale (standardized 20× objective lens magnification), as shown in Figure 2. The figure provides a comprehensive visualization of how wax incorporation alters the micro- and mesoscale surface structure of the coatings. The reference epoxy exhibits a relatively smooth and homogeneous morphology. Upon addition of waxes, distinct heterogeneous features emerge, characterized by dispersed domains, circular inclusions, and agglomerated structures distributed across the surface, as often seen elsewhere [36]. The introduction of micronized waxes in coatings is indeed known to change surface aspect and topography. Through a “floating” mechanism during film formation, these particles migrate to the coating surface and create micro-roughness, providing specialized surface aesthetics like tactile roughness (sand, suede, or suede textures), uniform matting, and high abrasion resistance [37]. At low wax content, the surface shows fine, evenly distributed microdomains, suggesting good dispersion of wax particles within the epoxy matrix. Increasing wax concentration results in the formation of larger, more irregular features and a higher density of protrusions, indicating phase separation and wax migration toward the surface. These effects are particularly pronounced for PE and HDPE systems, where large, well-defined domains and pronounced topographical peaks are observed. In contrast, RB wax exhibits a more uniform and finer dispersion of the wax phase, resulting in tiny surface structures [38].

**Figure 3:**
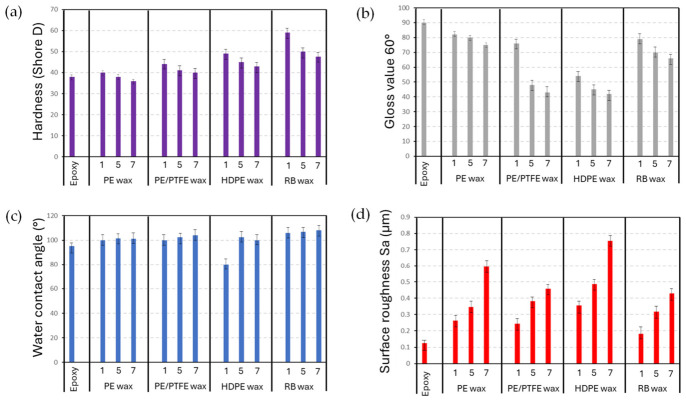
Intrinsic surface properties and mechanical properties (before laser patterning) of native epoxy coatings with different concentrations (1, 5, 7 wt.-%) of micronized wax additives, (**a**) hardness, (**b**) gloss, (**c**) water contact angle, (**d**) average surface roughness Sa.

The surface properties of the initial coatings are quantified in Figure 3, including measurements of surface hardness, gloss, water contact angles, and surface roughness. The data provides an overview of how wax additives systematically modify epoxy coating performance, in line with earlier studies [31]. Incorporation of PE-, PE/PTFE-, HDPE-, and RB waxes increases Shore D hardness relative to neat epoxy, reflecting the reinforcing effect of dispersed semicrystalline phases that restrict polymer chain mobility, as commonly observed for polyolefin-modified thermosets [39]. Simultaneously, gloss decreases with increasing wax content, which agrees with reports linking wax migration and surface micro-roughening to enhanced light scattering [40,41]. The increase in water contact angle confirms improved hydrophobicity, consistent with the introduction of low-surface-energy species and wax-induced surface structuring [42]. The higher average surface roughness values of Sa, especially for HDPE- and PTFE-containing systems, correlate well with both gloss loss and wetting behavior, reinforcing the established structure–property relationships for wax-modified epoxy coatings. As the wax microparticles precipitate, they indeed form fine particles that protrude from the surface. These protrusions reduce the contact area, reducing gloss (matting) and creating a textured feel with the given surface roughness and increased water contact angle.

The possible interactions of the wax particles with the epoxy matrix were identified in earlier studies [43] and may result in improvement in mechanical properties (hardness) at low concentrations, mainly for the presently selected small microparticle sizes. Further discussion on the influence of wax particles with different chemical compositions may be found elsewhere [31] and is not further elaborated here, as the collected data will mainly serve to establish influences of the coating properties on the laser patterning process.

### 3.2. Long-Range Evaluation of Laser Surface Texturing

**Figure 4:**
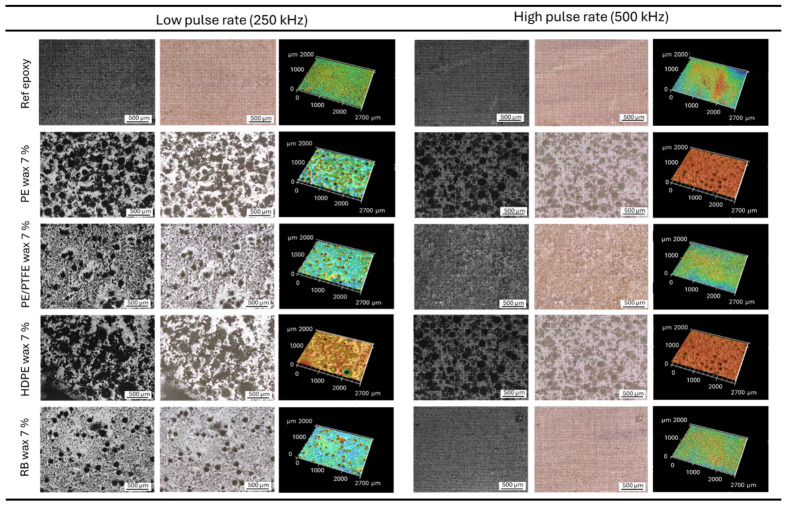
Long-range microscopic analysis of femtosecond laser-patterned epoxy coatings with different types of wax fillers (fixed concentration of 7 wt.-%) processed under a low pulse rate (250 kHz) and a high pulse rate (500 kHz) and constant 60 W laser power, including laser interference image, optical image, and 3D topographical image.

The feasibility for surface texturing of micronized wax-filled epoxy coatings through femtosecond laser patterning is first illustrated by long-range evaluation of the surface features, as demonstrated in Figure 4 for the selected coating compositions with pure epoxy coatings and epoxy coatings with 7 wt.-% of PE-, PE/PTFE-, HDPE-, and RB waxes. A similar analysis of all other coating compositions is included in Supplementary Information S2. The low pulse rates (250 kHz) and high pulse rates (500 kHz) were applied in combination with a fixed laser power setting of 60% in order to evaluate the effect of pulse rates on the reproducibility of surface patterns. The increase in pulse rate essentially determines the number of pulses that overlap per unit of time and affects the local heat accumulation. At the lower pulse rates, the pulses act more independently, and cooling of material between the pulses is allowed, while there is strong pulse overlap and heating accumulation at the higher pulse rates.

For the pure epoxy coatings, homogeneous patterning over a long-range area is observed at both low and high pulse rates. The epoxy matrix indeed represents a thermoset with a 3D crosslinked network with high stability. The material is not subject to true melting and may start to decompose rather than flow. The epoxy material retains a much higher viscosity as compared to the wax, even when heated; therefore, the melt flow and reorganization of the epoxy coating are suppressed. Under the input of laser energy, the ablation likely occurs before melting can develop. Fundamental understanding of the mechanisms of epoxy under femtosecond laser drilling has been described [44]. The epoxy might be prone to degradation by breaking of chemical bonds through photochemical and multiphoton reactions while the material directly goes from a solid into a gas or plasma phase (the so-called “cold ablation” process dominates). As a result, the pulse repetition rates do not strongly affect the epoxy matrix, as no melting is created at low repetition rates and the heat does not build up enough to cause melting or flow at the high pulse repetition rates. Moreover, the localized heating in the epoxy matrix at high repetition rates does not cause melting and a local drop in viscosity, ensuring a stable pattern geometry. This is different compared to the laser processing of polymers in the microsecond time scales [45]. It can be assumed that the plasma effects during femtosecond laser patterning in epoxy are less disruptive as the interaction time is very short (femtosecond range) and the ablation plume is less dense due to the accumulation of fragmented products [46]. Hence, the epoxy shows progressive incubation of effects that progressively accumulate during the ablation process while the pulse-to-pulse interferences are minimized. As a result, the laser ablation indeed results in spatial uniformity of the patterned surface. Similarly, a good reproducible fine granular morphology could be produced in the epoxy matrix of fiber-reinforced composites [47].

For the wax-filled coatings, the patterning indicates irregular surfaces with visual melting defects (e.g., melt rims, re-deposited droplets, flow lines, and irregular topography) at low pulse rates and more homogeneous patterning at higher pulse rates. This might occur as a counterintuitive effect in laser processing of low-melting, low-thermal-conductivity materials, like waxes and thermoplastic polymeric coatings. In heat-sensitive materials, such as waxes with low melting temperature, low boiling point, low thermal conductivity, low viscosity when molten, and weak structural stability, the balance between heat accumulation and heat dissipation becomes critical. Hence, the melting threshold is much lower than the ablation threshold, and small heat accumulation leads to fluid behavior of waxes. At low pulse repetition rates, each pulse deposits energy into a relatively “cold” material with time for heat to diffuse and spread laterally, promoting bulk melting. Because the temperature rise per pulse is moderate, the melting temperature of waxes can be easily reached but does not strongly exceed the boiling/decomposition point. As the melt lifetime between the pulses is relatively long (µs-scale), the wax viscosity locally drops significantly and leads to the accumulation of liquid material. Therefore, stable melt pools are formed while the melt can grow and spread. At high pulse repetition rates, in contrast, rapid energy build-up drives fast vaporization and material ejection, leaving less time and volume for stable melt formation. With the intense and very localized heat accumulation at high pulse rates (i.e., “the next pulse arrives before cooling”), the local temperature likely rises above the wax boiling point of the wax, causing immediate material removal through ablation/evaporation before the melting regime is settled. The strong plume formation results in melt explosion and surface cleaning of residual molten deposits.

The present experiment supports unique data on laser patterning of wax-filled coatings and the need for very precise control of the processing parameters in relation to the wax. The laser ablation of wax-based superhydrophobic coatings has only been very recently applied in relation to paper coatings [48]; however, it demonstrated the selective removal or modification of wax layers while preserving substrate integrity. In conclusion from our work on wax-filled coatings, the energy couples mainly into heating and melting at low pulse rates, while the interaction becomes dominated by rapid vaporization, ejection, and poor energy coupling to the melt at high pulse rates. Consequently, the homogeneity in femtosecond laser patterning depends on whether the morphology is melting-dominated (phase-change) at low pulsation rates or ablation-dominated (bond breaking) at high pulsation rates.

### 3.3. Detailed Evaluation of Laser Surface Texturing

**Figure 5:**
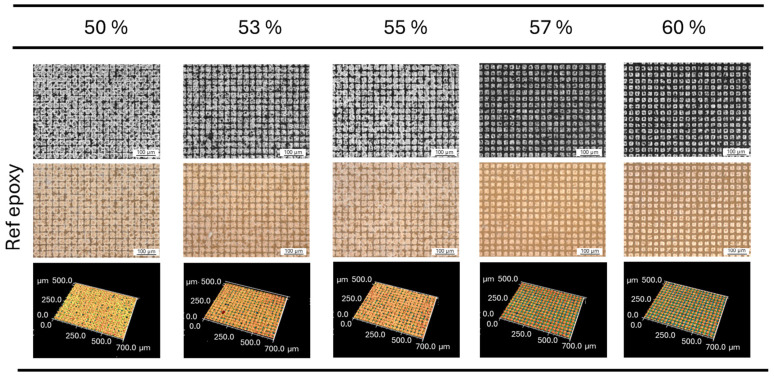
Detailed microscopic and topographical analysis of femtosecond laser-patterned epoxy reference coatings at different laser power settings (50 to 60%) and a pulse rate of 500 kHz, including laser interference image, optical image, and 3D topographical image.

**Figure 6:**
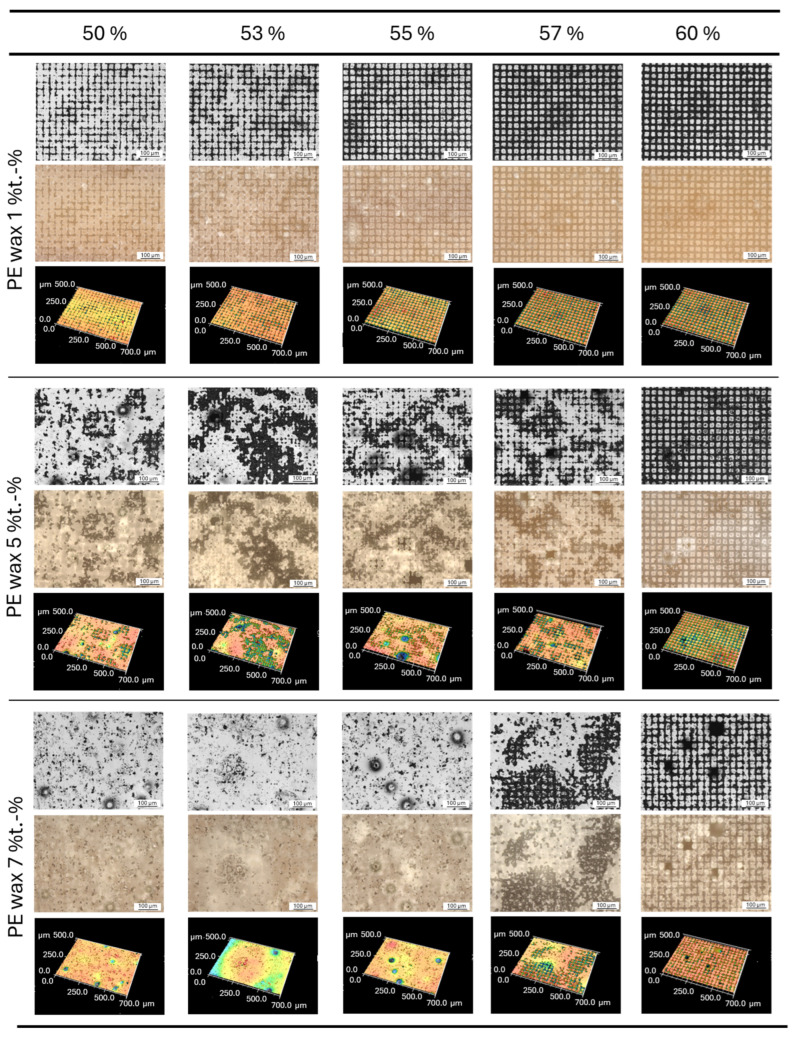
Detailed microscopic and topographical analysis of femtosecond laser-patterned epoxy coatings with different concentrations of PE wax at different laser power settings (50 to 60%) and a pulse rate of 500 kHz, including laser interference image, optical image, and 3D topographical image.

**Figure 7:**
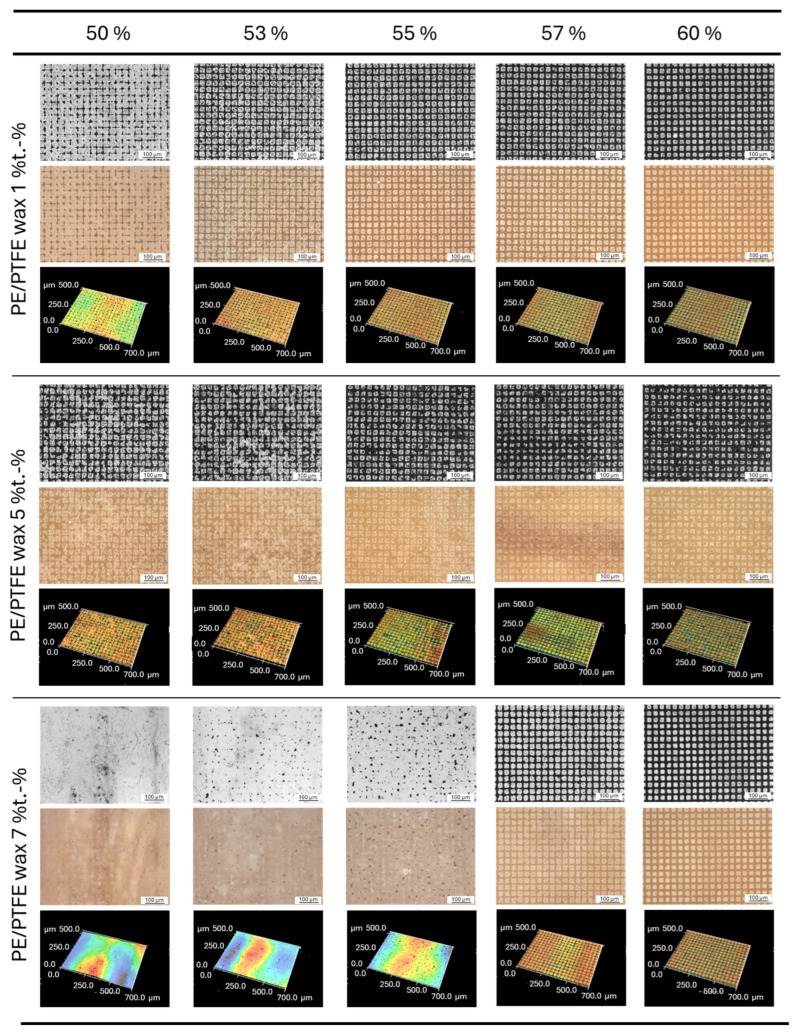
Detailed microscopic and topographical analysis of femtosecond laser-patterned epoxy coatings with different concentrations of PE/PTFE wax at different laser power settings (50 to 60%) and a pulse rate of 500 kHz, including laser interference image, optical image, and 3D topographical image.

**Figure 8:**
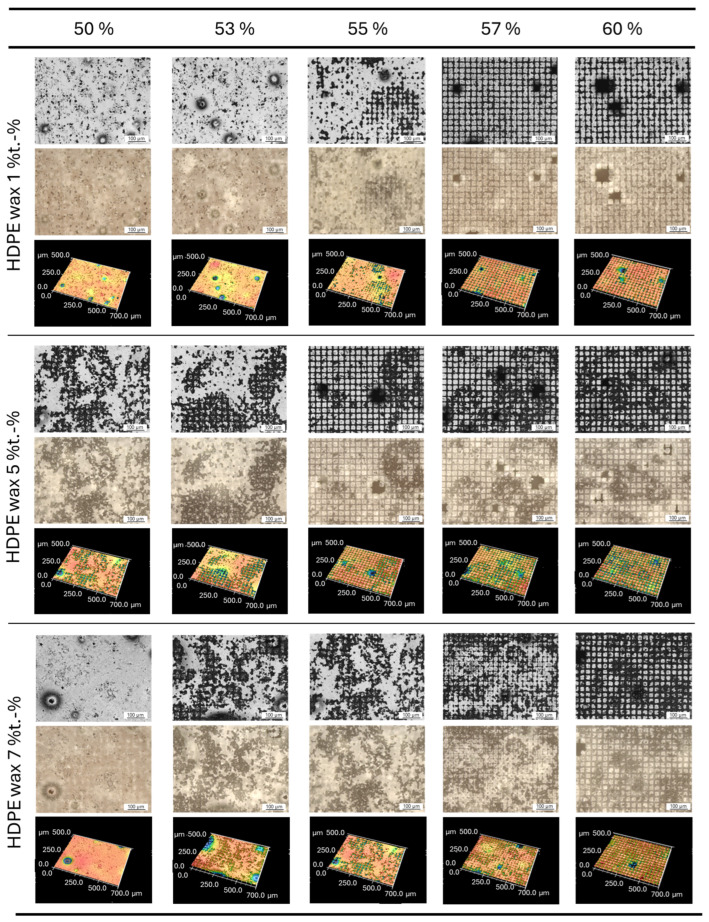
Detailed microscopic and topographical analysis of femtosecond laser-patterned epoxy coatings with different concentrations of HDPE wax fillers at different levels of laser power, including laser interference image, optical image, and 3D topographical image.

**Figure 9:**
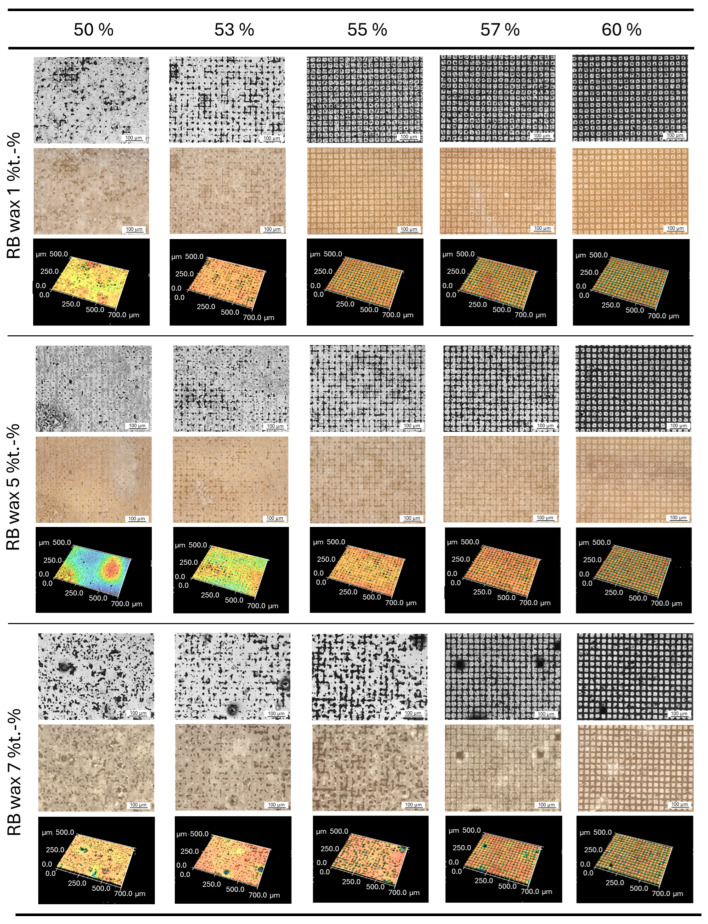
Detailed microscopic and topographical analysis of femtosecond laser-patterned epoxy coatings with different concentrations of RB wax fillers at different levels of laser power, including laser interference image, optical image, and 3D topographical image.

The feasibility for surface texturing of epoxy reference coatings and epoxy coatings with different types and concentrations of micronized wax additives is illustrated in Figure 5, Figure 6, Figure 7, Figure 8 and Figure 9 for the reference epoxy coatings (Figure 5) and filled epoxy coatings with PE wax (Figure 6), PE/PTFE wax (Figure 7), HDPE wax (Figure 8), and RB wax (Figure 9).

The images demonstrate the effect of a gradual increase in laser power setting between 50 to 60% on the creation of a homogeneous surface pattern while applying a constant hatch pitch of 35 µm and a pulse rate of 500 kHz. The increase in laser power introduces locally higher energy densities with different effects on the epoxy coatings and wax-filled epoxy coatings. As derived from the 3D topographical scans (20× standard magnification), the respective increase in average surface roughness Sa of the patterned surfaces processed at increasing laser power settings is quantified in Supplementary Information S3.

For the pure epoxy coatings, the increasing laser power evidently shifts the patterning from a dense, fine, somewhat noisy texture at low power towards the emergence of regular periodic structures at intermediate power and a clean pattern geometry with very sharp, high-contrast, well-separated square features at high power. The creation of a well-defined patterning texture is related to the ablation threshold, above which efficient ablation occurs. Efficient incubation and ablation occur through stable pulse-to-pulse material removal with minimized thermal distortion, but only for the laser fluences close to the ablation threshold. The laser fluences below the ablation threshold only cause limited material removal in parallel with the weak incubation and limited energy for breaking of the crosslinked polymer network. The high laser fluences resulting in very sharp patterns introduce strong material ablation per pulse and efficient material removal [49]. At higher power, there might be slight heat accumulation in parallel with the minor thermal distortion of the epoxy matrix without melting or pattern distortion [50]. In conclusion, the increasing laser power in femtosecond patterning of epoxy progressively drives the system from near-threshold modification to efficient, highly uniform ablation, resulting in deeper, sharper, and more defined periodic structures—without the instability associated with melt-driven materials.

For the wax-filled epoxy coatings, it is counterintuitively observed that the higher laser power (energy input) does not provide better pattern geometries, in contrast with the occurrence of irregular melting patterns at the lower laser power. This is a typical effect in laser processing of low-melting materials, like wax, and the explanation relates to how the absorbed energy is dissipated rather than how much is delivered [51]. At a low laser power, energy is primarily used to heat, and the temperature increases gradually. Hence, the absorbed energy goes into sensible heating and latent heat of fusion (melting) of the wax, allowing the wax melt to accumulate and spread. At a high laser power, local temperatures rise very rapidly above the boiling point and thermal decomposition point, resulting in rapid vaporization and material ejection; so, the melt phase is short-lived and less visible. The higher laser power does not necessarily mean more melting—beyond a certain point, increasing power shifts the process from controlled melting to rapid material removal, preventing the melt from forming or persisting.

**Figure 10:**
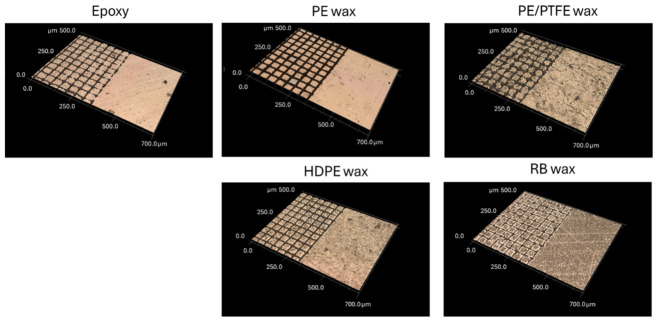
Detailed microscopic analysis of femtosecond laser-patterned epoxy coatings with 7 wt.-% of different wax fillers and femtosecond laser patterning conditions at ablation threshold, indicating the transition between patterned and non-patterned surface area with better observation of the irregularities on top of the posts.

For the selected parameters during femtosecond laser processing of the different coatings near the ablation threshold conditions (500 kHz, variable power setting corresponding to the laser ablation threshold), a detail at the border between patterned and non-patterned zones with further morphological details on top of created spots is shown in Figure 10. In particular, the material accumulation may be detected as a result of the debris deposition created in the plasma plume. The latter is most prevalent for the PE/PTFE wax deposits, followed by the RB wax deposits. While these observations do not directly correlate with the thermal stability of the wax in terms of their melting temperature *Tm*, the melting enthalpy *ΔH* of the respective waxes may be a better indicator. The relations are further elaborated in the next paragraph, relating ablation threshold with intrinsic wax properties.

### 3.4. Ablation Threshold and Intrinsic Coating Properties

**Figure 11:**
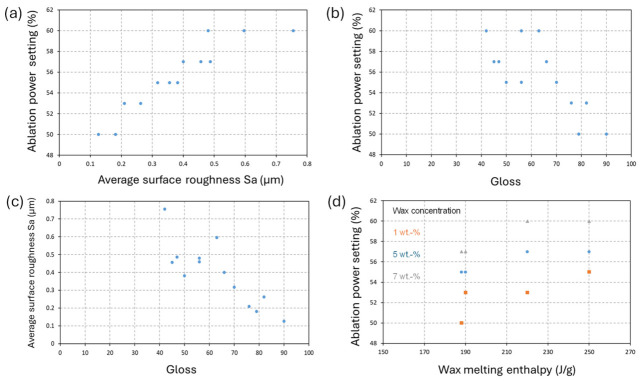
Correlation of the ablation threshold (here expressed as the ablation power at 500 kHz) with the intrinsic coating properties, including (**a**) average surface roughness Sa, (**b**) gloss, (**c**) relation between gloss and average surface roughness, (**d**) wax melting enthalpy for different wax concentrations.

After determination of the ablation threshold values for laser surface patterning of the coatings at laser power settings between 50 and 60% (see Supplementary Information S4), the possible relationships between ablation threshold and intrinsic coatings or material properties were mapped (Figure 11), including the influences of roughness and gloss (Figure 11a–c) or wax melting enthalpy (Figure 11d).

For the present polymeric coatings, the observed trend for the increase in ablation threshold values with roughness (Figure 11a)—and, consequently, the decrease in ablation threshold with gloss (Figure 11b) given the unique correlation between surface gloss and roughness (Figure 11c)—is different from the traditional roughness–threshold relationship that is expected for femtosecond laser patterning of hard and reflective surfaces (e.g., metal, ceramics). For conductive or strongly absorbing materials, a smooth surface would introduce higher reflectivity and less absorbed energy, while a rough surface would introduce multiple scattering effects and more absorption [52]. These physical phenomena result in a reduction in the ablation threshold on the rougher surfaces. In contrast, the reversed trend for the polymer coatings in the present study may come from the fact that, in polymers under femtosecond irradiation, optical absorption is rarely governed purely by Fresnel reflectivity or geometric surface area. Instead, subsurface nonlinear absorption, defect states, and thermal–mechanical responses might dominate the interaction with the laser beam [53]. Most polymers are transparent or weakly absorbing at common femtosecond wavelengths (e.g., 800–1030 nm), while the ablation is initiated by multiphoton absorption and avalanche ionization [54]. Therefore, this shifts control of material versus beam interaction from surface optics to bulk electronic processes. Possible effects on the rough polymer surfaces may include light scattering away from the focal volume, resulting in a reduction in the peak intensity at the focal spot and beam distortion. As a result, the absorption of laser energy at the focal volume is less efficient. Alternatively, the smoother and glossy polymer surfaces may better preserve the beam quality and minimize the wavefront distortion, resulting in a higher peak intensity in the focusing area. As the energy dissipation in polymers under femtosecond laser processing occurs below the surface rather than on the top surface, it is driven by nonlinear absorption within the focal volume [55]. The introduced surface roughness disrupts these interactions by locally changing local incidence angles, introducing micro-defocusing points, and creating spatial intensity fluctuations. In conclusion, for the polymeric surface coatings, ablation might be governed by peak intensity-driven nonlinear absorption in the focal volume, rather than by surface reflectivity or geometric absorption.

Most interestingly, the positive correlation between ablation power and wax melting enthalpy ΔH_m_ for the coatings with different wax concentrations (Figure 11d) is observed, with standard reference values (as provided by distributor) of ΔH_m_ = 188 J/g (PE/PTFE wax), ΔH_m_ = 190 J/g (RB wax), ΔH_m_ = 220 J/g (PE wax), and ΔH_m_ = 250 J/g (HDPE wax). In particular, for the RB wax and PE/PTFE wax with the lowest melting enthalpy, better reproducibility of the surface patterns has been observed at the different laser powers. Alternatively, no direct correlation was found with the melting temperatures *T_m_* of the waxes, with *T_m_* = 78 °C (RB wax), *T_m_* = 110 °C (PE wax), *T_m_* = 115 °C (PE/PTFE wax), and *T_m_* = 128 °C (HDPE wax). This confirms that the femtosecond laser patterning of the wax-filled coatings happens in a thermally controlled melting regime. In femtosecond ablation of thermoplastics, material removal often proceeds via superheating of the molten phase and phase explosion/spinodal decomposition [56,57,58]. If ΔH_m_ is high, more laser fluence (energy) is needed just to generate the molten wax phase, and less energy remains (initially) for superheating. Therefore, the increase in threshold ablation power with the higher melting enthalpy of the wax is fundamentally tied to how much energy is required to drive the material through the phase transitions and ultimately into material removal under ultrafast conditions. The melting transition of waxes is a necessary intermediate step before material removal becomes efficient. For the materials with a higher melting enthalpy, more of the introduced laser energy is consumed in overcoming the melting transition (internal energy storage in the material as latent heat), leaving less energy available for the rapid superheating and mechanical disintegration that drive ablation.

### 3.5. Water Contact Angles and Hydrophobicity Modeling

**Figure 12:**
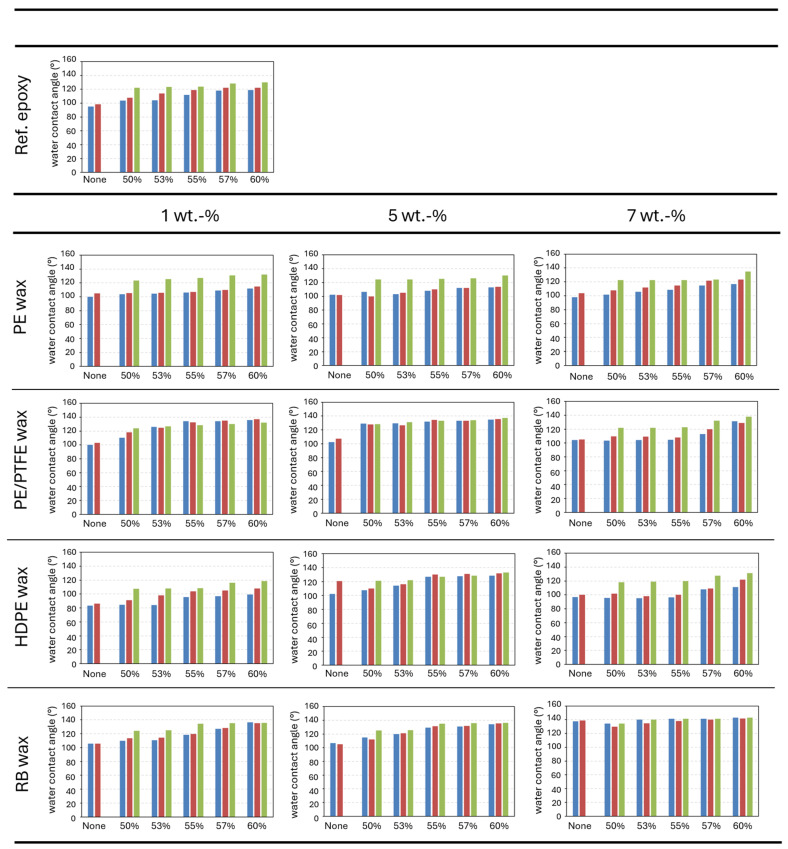
Experimental measurements and calculated model data of static water contact angles on reference epoxy coatings and wax-filled epoxy coatings with 1, 5, and 7 wt.-% of PE wax, PE/PTFE wax, HDPE wax, and RB wax before and after femtosecond laser patterning at different laser power settings (50 to 60%) (see X-scales, with “None” indicating the non-patterned coatings), including experimental value after patterning (blue bar), experimental value after additional thermal curing (red bar), and theoretical value calculated according to the Cassie–Baxter model (green bar).

With the aim of increasing the hydrophobic properties of the femtosecond laser-patterned coatings, the static water contact angles were experimentally measured on the non-patterned coatings and after patterning at various laser powers (fixed geometries with a hatch pitch of 35 µm and a pulse rate of 500 kHz). The repeatability of the water contact angles was checked immediately after laser patterning (Figure 12, blue bars) and after an additional thermal treatment for 30 min at 60 °C, remaining below the melting temperature of the wax (Figure 12, blue bars), and it was applied in order to homogenize the top surface and control eventually migrated wax. In addition to the summary of contact angle data in Figure 12, selected contact angle images on native coatings and after laser texturing at a 60% power setting are documented in Supplementary Information S5. The variations between the contact angles directly after laser patterning and with additional thermal treatment are statistically insignificant, demonstrating good reproducibility of the measurements with significant trends. Obviously, no additional thermal effects stimulating hydrophobicity through eventual migration of the wax are introduced after laser patterning. Mainly, the differences between the two measuring conditions decrease and nearly overlap at the higher laser powers, indicating that surface morphology dominates wetting behavior. The increasing laser power reduces the sensitivity to the specific measurement condition, likely due to better surface homogeneity and dominant surface structuring effects. As it was confirmed that the original epoxy coatings before laser patterning were hydrophobic, it can be noticed that the patterning at higher laser power introduced an increase in hydrophobicity to different extents depending on the initial water contact angle and perfection of the pattern geometry.

For the pure epoxy coatings, the relatively low water contact angle on the non-patterned coatings, in parallel with the gradually sharpened patterns as a function of higher laser power, introduces a gradual increase in water contact angles towards moderate values. For the epoxy coatings with PE wax, the irregular surface patterns due to melting phenomena at low laser power, as explained before, do not significantly improve the hydrophobicity of the coatings, except for a significant increase in water contact angle at the highest laser power setting of 60%, in parallel with the better resolved surface patterns. The fact that water contact angle measurements after laser patterning (blue bar) and additional thermal treatment (red bar) have little variation confirms that the thermal melting might have occurred during laser texturing. Similarly, for the coatings with PE/PTFE wax at 1 to 5 wt.-%, the progressively better resolved surface patterns at higher laser power are in line with the gradual improvement in water contact angles. In parallel, the previous melting-induced processing of PE/PTFE wax coatings at 7 wt.-% as explained before results in weak patterning at low power and no increase in hydrophobicity. For the epoxy coatings with HDPE wax, the irregular features of the patterned surfaces and the moderate hydrophobicity of the native coatings, however, provide a limited increase in water contact angles. The system requires higher laser power to fully develop better homogeneity of the laser patterns and, consequently, higher hydrophobicity. For the epoxy coatings with RB wax at 1 to 5 wt.-%, a most favorable increase in hydrophobicity is noticed in parallel with the relatively high initial hydrophobicity of the non-patterned coatings and the high reproducibility of the surface patterns. For the coatings with RB wax at 7 wt.-%, the coating presents nearly constant high water contact angles. With the increase in laser power, the effect of laser structuring on hydrophobicity saturates, and water contact angles have already maximized at moderate power. As the latter coatings already have very high intrinsic hydrophobicity and laser patterning provides little added benefit, surface chemistry likely starts dominating over morphology already at low laser power.

In conclusion, within the present set of experimental data, a maximized water contact angle of 143° was obtained for the epoxy coatings with 7 wt.-% RB wax after laser patterning at a 60% power setting and a 500 kHz pulse rate relative to a contact angle of 137° on the non-patterned substrate (4.4% increase due to patterning). Alternatively, the epoxy coatings with 1 wt.-% RB wax presented a water contact angle of 137° after laser patterning at a 60% power setting and a 500 kHz pulse rate relative to a contact angle of 106° on the non-patterned substrate (29.3% increase due to patterning).

The theoretical values of water contact angles on patterned coatings (Figure 12, green bars) were approximated through calculations following the Cassie–Baxter model under the form in Equation (1) as motivated before for the given geometry of island-like patterns [59]:

cos *θ_c_* = *r*_1_ *f_s_* cos *θ_s_* − (1 − *f_s_*) (1)

For the island-like geometries with post sizes, including height H (µm), top length T (µm), and valley depth B (µm), the roughness parameter *r*_1_ is defined as in Equation (2), and the fraction of the solid surface area in direct contact with the liquid *f_s_* is defined as in Equation (3). We refer to our previous publication for the motivation of the selected parameters and the influence of the different parameters H, B, and T on the apparent contact angle *θ_c_* in relation to the equilibrium contact angle *θ_s_* on a non-patterned solid surface [60]. With the present selection of pattern geometry with a hatch pitch of 35 µm, the values B = 15 µm and T = 20 µm were set, and height H was taken from 2D dimensional topographical scans.

*r*_1_ = (T + B)/(2H + T + B) (2)

*f_s_* = T/(T + B) (3)

**Figure 13:**
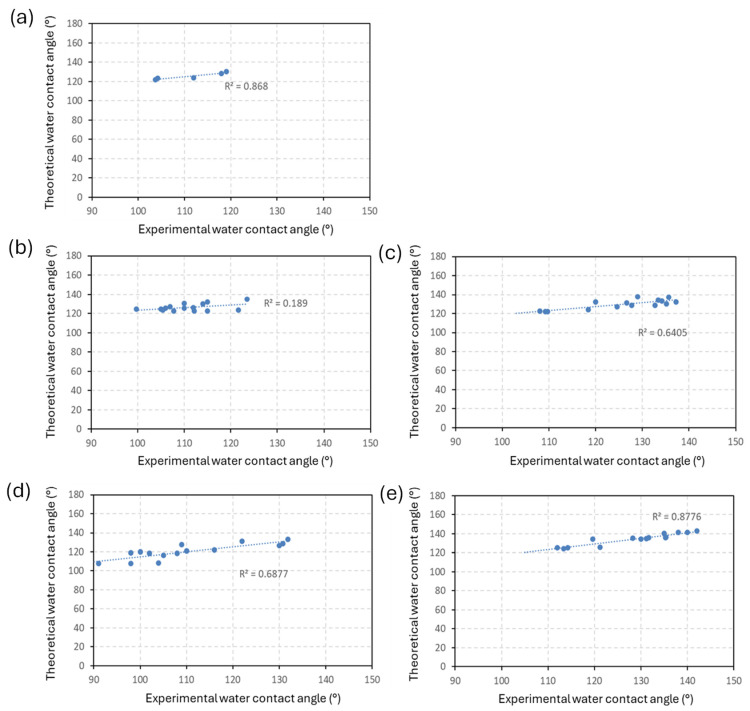
Correlation between experimental contact angles and theoretically calculated contact angles (Cassie–Baxter) after femtosecond laser texturing of different coatings, including (**a**) ref. epoxy coating, (**b**) epoxy coating + PE wax at different concentrations, (**c**) epoxy coating + PE/PTFE wax at different concentrations, (**d**) epoxy coating + HDPE wax at different concentrations, (**e**) epoxy coating + RB wax at different concentrations.

On the contrary, the poor correlations between experimental water contact angles and the average surface roughness Sa of the coatings after femtosecond laser patterning are illustrated in Supplementary Information S6. As no direct correlation appears between the apparent contact angle *θ_c_* and the equilibrium contact angle *θ_s_* or average surface roughness Sa, the simple model for Wenzel wetting with cos *θ_c_* = r cos *θ_s_* (roughness parameter *r*) could not be validated. The calculations according to the latter model consequently result in lower theoretical values than the presented experimental values for apparent contact angles. A better proof for the observed trends on the relation between theoretical water contact angles according to the Cassie–Baxter model with roughness parameter *r*_1_ and experimental values on homogenized laser-patterned substrates (i.e., after thermal treatment for 30 min at 60 °C) is given in Figure 13 for the different coating types. Cassie–Baxter wetting refers to a metastable state in a composite wetting regime with air entrapment in the surface pockets below the water droplet; however, it is evidently lost with increasing volume of the water droplet (increase in pressure) or with external vibration (Cassie-to-Wenzel transition), as illustrated in Supplementary Information S7.

Taking into account that the calculation results based on the Cassie–Baxter model assume the effect of surface texturing on chemically homogeneous substrates, differentiations between the experimental and theoretical results may be attributed to the chemically heterogeneous nature of the wax-filled epoxy coating. Nevertheless, the representative trends are observed with variable statistical significance depending on the type of coating. The latter indeed highly depends on the homogeneity of the surface patterns that were applied on the coating under different laser power, i.e., best correlations are observed for the reference epoxy coatings (R^2^ = 0.86) and the coatings with RB wax fillers (R^2^ = 0.88). Hence, the absence of significant thermal melting of RB wax in contrast with PE, PE/PTFE, and HDPE waxes results in more reproducible data. In conclusion, the higher perfection of the pattern geometries on RB wax-filled coatings results in a close correlation between experimental water contact angles and theoretical calculations. As before, the better reproducibility of the patterns is in line with the lower melting enthalpy and is expressed as lower average surface roughness Sa of epoxy coatings with RB wax.

### 3.6. Chemical Surface Analysis (FTIR Spectroscopy)

**Figure 14:**
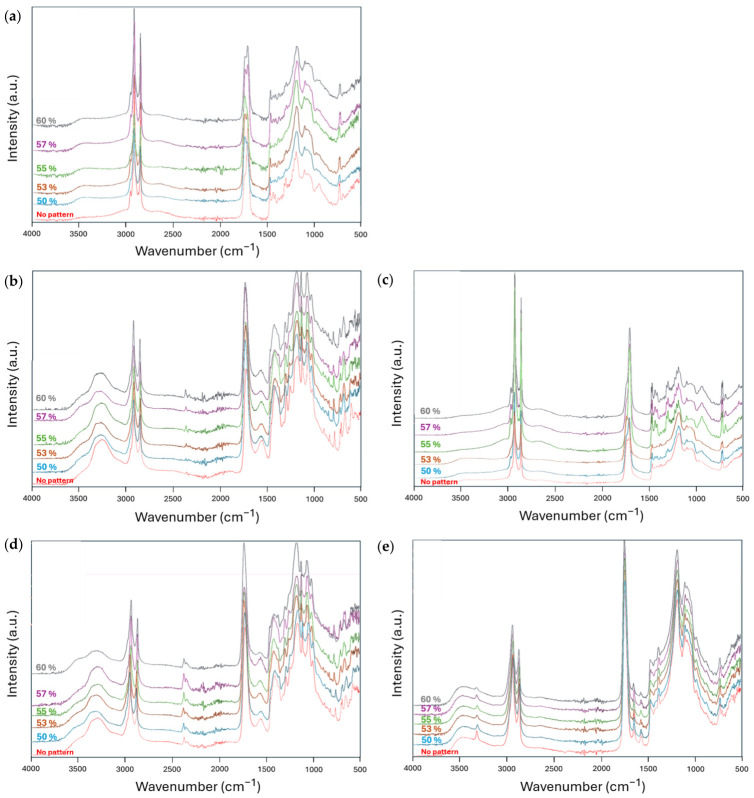
Fourier-transform infrared (FTIR) spectra of different coatings before and after femtosecond laser patterning at different laser power settings (50 to 60%), including (**a**) native epoxy coating, (**b**) epoxy + 5 wt.-% PE wax, (**c**) epoxy + 5 wt.-% PE/PTFE wax, (**d**) epoxy + 5 wt.-% HDPE wax, (**e**) epoxy + 5 wt.-% RB wax.

The FTIR spectra of different coatings before and after laser surface texturing at different power settings (50 to 60%) are shown in Figure 14, indicating the eventual chemical modification and degradation at the top surface.

The spectra of pure epoxy coatings (Figure 14a) confirm the composition of a crosslinked vegetable oil with acids [61], including the spectral features related to alkyl chains (fatty acid backbone) at 2925–2850 cm^−1^ (–CH_2_–, –CH_3_ stretching), 1465–1440 cm^−1^ (CH_2_ bending), and 1375 cm^−1^ (CH_3_ bending); ester groups (triglyceride structure + crosslinking) at 1740 to 1730 cm^−1^ (C=O stretching of an ester carbonyl) and 1240–1160 cm^−1^ (C–O–C stretching of ester); carboxylic acids (curing agent or residuals) at 2500–3300 cm^−1^ (O–H stretch of H-bonded acid) and 1710–1690 cm^−1^ (C=O stretching in COOH, slightly lower than ester); hydroxyl groups (formed after epoxy ring opening) at 3200–3600 cm^−1^ (O–H stretching); and ether linkages at 1100–1050 cm^−1^ (C–O–C stretching of secondary alcohols/ethers). The absence of the epoxy ring vibrations at 915–910 cm^−1^ (epoxide ring breathing) and residual unsaturation at 1650–1600 cm^−1^ (C=C stretching) confirms a successful epoxidation reaction after curing. As a function of increasing laser power setting, peak intensity decreases, and slight broadening or distortion happens at around the ester carbonyl bond (1740 cm^−1^) as an indication for chemical cleavage of the ester bond through photochemical reactions with possible formation of CO_2_ and smaller oxidized fragments [62]. The increase in carbonyl moieties (1710 cm^−1^) is an indication of oxidative degradation and formation of ketones, aldehydes, and carboxylic acid fragments [63,64]. However, oxidation and surface activation remain limited as there is no significant increase in the free hydroxyl group region (3600 to 3000 cm^−1^). Alternatively, no variations in the epoxy functionalities (oxirane group), the aliphatic chains (linear fatty acid chains), and fingerprint area (1500–1000 cm^−1^) are observed after laser texturing. In particular, band broadening, baseline distortion, and loss of sharp molecular features typically observed after a carbonization reaction do not occur. The spectra confirm a limited thermal accumulation during femtosecond laser pulsing while avoiding carbonization and the formation of amorphous carbon-like char deposits. In conclusion, the laser texturing of pure epoxy coatings mainly introduces slight fragmentation of the triglycerides originating from the epoxidized oil.

The spectra of wax-filled epoxy coatings (Figure 14b–e) have introduced the presence of wax mainly through the occurrence of aliphatic bands at 2925–2850 cm^−1^ (–CH_2_–, –CH_3_ stretching), which overlap with the composition of the epoxy matrix [65]. In particular, the RB wax is an ester-rich natural wax, while PE waxes are richer in linear hydrocarbons [66]. Therefore, exact quantitative analysis cannot be done, but a qualitative interpretation of the spectra does not indicate noticeable migration effects of wax towards the surface after laser patterning, given the constant relative intensity of the related spectral bands of aliphatic chains. Moreover, the better chemical stability after laser texturing of the epoxy matrix in coatings with PE, HDPE, and RB wax compared to the pure epoxy coatings is observed, given the constant shapes and intensity of the 1740 to 1730 cm^−1^ band. Some more pronounced degradation in the epoxy coatings with PE/PTFE fillers is seen at around the 1740 to 1730 cm^−1^ band, likely due to the more stable molecular structure of the PTFE with strong chemical bonds, higher thermal stability, and higher decomposition temperature that does not provide enough shielding of the matrix through uptake of the laser energy. As mentioned before, the other wax types may protect the epoxy matrix from exposure to degradation through partial uptake of the laser energy through melting, depending on their melting enthalpy. In that respect, the epoxy coatings with bio-based RB wax show the highest structural stability after laser processing, which may be related to the fact that it is an amide wax type (see bands at 3300 cm^−1^, 1650 cm^−1^, and 1530 cm^−1^). The latter seems to be chemically more reactive under laser processing as compared to the aliphatic wax types, given the reduction in amide moieties through additional crosslinking after laser processing. Therefore, the superior performance of functionalized waxes (although proprietary grades) in terms of laser texturing, chemical stability, and hydrophobicity is confirmed in this study.

## 4. Conclusions

This study has demonstrated the feasibility and governing mechanisms of femtosecond laser surface texturing and enhancing the hydrophobicity of bio-based epoxy coatings with and without the incorporation of micronized wax additives. Therefore, epoxy coatings were formulated with different types and concentrations of fossil- and bio-waxes, followed by the laser texturing under different conditions of pulse rates and laser power settings. The innovative aspect encompasses a combined approach of surface chemistry and surface topography in order to tune the hydrophobic performance of the soft polymer coatings, where the selection of appropriate laser processing parameters can be related to the intrinsic coating properties.

A key finding is the strong correlation between ablation threshold and intrinsic coating properties. In particular, ablation resistance increases with surface roughness and with the melting enthalpy of the incorporated waxes, indicating that energy consumption during phase transitions plays a decisive role in ultrafast laser processing. Due to the low melting temperatures of waxes, the coatings exhibit a transition from melting-dominated behavior at low pulse rates to ablation-dominated behavior at higher pulse rates.

Femtosecond laser texturing of wax-filled epoxy coatings enhances hydrophobicity, with the extent of improvement depending on the initial coating composition and the quality of the generated patterns. Based on present experimental data, a maximum water contact angle of 143° was obtained on epoxy coatings with 7 wt.-% RD wax. The highest water contact angles occur on coatings combining intrinsic hydrophobicity with stable pattern formation, such as those with rice bran wax. The experimental trends are reasonably described by the Cassie–Baxter model, while expected deviations arise from chemical heterogeneity and morphological imperfections in melt-affected systems. Chemical analysis confirms that femtosecond laser processing induces only limited degradation of the epoxy matrix, with no evidence of severe carbonization. Wax additives may provide partial thermal shielding depending on their phase transition characteristics (melting enthalpy), further contributing to coating stability.

In general, the importance of controlling energy input and the melting behavior of the waxes in pattern generation has been demonstrated. Therefore, future work should focus on further reducing melting influences to increase the quality of laser patterns through, e.g., an increase in pulse rates (1000 to 2000 kHz), the use of laser features, such as burst mode to achieve GHz pulses, or the use of a compressor to decrease femtosecond pulse duration down to 50 fs. The latter depends on the availability of appropriate laser processing technology. Moreover, the mechanical and environmental durability of the resulting coatings should be further investigated.

## Figures and Tables

**Table 1:** Operational conditions for femtosecond laser patterning at a laser power setting of 50 to 60%, a pulse rate of 250 and 500 kHz, and a spot size diameter of d = 17 µm.

Laser Power Setting		50%	53%	55%	57%	60%
Pulse rate 250 kHz	Pulse energy [µJ]	8.91	10.6	11.8	13.1	15.0
	Laser fluence [J/cm^2^]	5.04	6.02	6.71	7.41	8.52
	Distance between spots, D [µm]	16				
	d/D	1.06				
Pulse rate 500 kHz	Pulse energy [µJ]	4.45	5.31	5.92	6.54	7.52
	Laser fluence [J/cm^2^]	2.52	3.01	3.35	3.71	4.25
	Distance between spots, D [µm]	8				
	d/D	2.13				

## Data Availability

The dataset is available upon request from the authors.

## Supplementary Materials

The following supporting information can be downloaded at: https://www.mdpi.com/article/10.3390/mi17060759/s1, Figure S1. Microscopic picture of preliminary experiments when laser texturing outside the operational window of power setting, (a) <50% power setting, (b) >60% power setting; Figure S2. Full dataset for long-range microscopic analysis of femtosecond laser-patterned epoxy coatings with different types of wax fillers (fixed concentration of 7 wt.-%) processed under a low pulse rate (250 kHz) and a high pulse rate (500 kHz) and constant 60 W laser power, including laser interference image, optical image, and 3D topographical image; Figure S3. Average surface roughness Sa (µm) on reference epoxy coatings and wax-filled epoxy coatings with 1, 5, and 7 wt.-% of PE wax, PE/PTFE wax, HDPE wax, and RB wax before and after femtosecond laser patterning at different laser power settings (50 to 60%) (see X-scales, with “None” indicating the non-patterned coatings); Figure S4. Determination of ablation threshold value for different coatings at 500 kHz; Figure S5. Illustration for static water contact angles on native coatings (before laser texturing) and after laser texturing at 60% power setting, 500 kHz, and 35 µm pitch distance, with indication of representative numerical value; Figure S6. Direct relation between experimental measurements of water contact angles and average surface roughness Sa, including reference epoxy coatings and wax-filled epoxy coatings with 1, 5, and 7 wt.-% of PE wax, PE/PTFE wax, HDPE wax, and RB wax before and after femtosecond laser patterning at different laser power settings (50 to 60%); Figure S7a. Variations of water contact angles onto a laser-patterned epoxy coating with 7 wt.-% RB wax depending on the water droplet volume and external vibration, with a transition from Cassie–Baxter wetting into Wenzel wetting at the higher droplet volume and with the application of external vibration; Figure S7b. Time-lapse frames for the deposition of a water droplet (1 µL) on a laser-textured epoxy coating with 7 wt.-% RB wax, with a microscopic detail of the textured surface morphology.
