# Supplementary material for: Femtosecond Laser Texturing of Wood Coatings with Bio-Based Epoxy and Wax Additives for Enhanced Hydrophobicity
Source: Micromachines (Basel). 2026 Jun 22;17(6):759. doi: 10.3390/mi17060759 (PMC13303751; doi:10.3390/mi17060759)
Supplement: Supplementary file 1 [file micromachines-17-00759-s001.zip › micromachines-4343782-supplementary.pdf]

micromachines

Article

Femtosecond Laser Texturing of Wood Coatings with Bio-Based Epoxy and Wax Additives for Enhanced Hydrophobicity

Pieter Samyn [1,*], Patrick Cosemans [1] and Olivier Malek [2]

[1] Department of Innovations in Circular Economy and Renewable Materials, SIRRIS, 3001 Leuven, Belgium; pieter.samyn@sirris.be, patrick.cosemans@sirris.be
[2] Department of Manufacturing Systems and Technologies, SIRRIS, 3600 Genk, Belgium; olivier.malek@sirris.be
* Correspondence: pieter.samyn@sirris.be

Supplementary information S1.

Illustration of maleficent laser surface patterning of the coatings at power ranges outside the operational window at power settings below 50 % and above 60 % is provided in Figure S1, showing that either no patterning has been observed or defective structures are created. At < 50% power, laser fluence would fall significantly below the ablation threshold of the coatings. Expected effects include a weak energy coupling via multiphoton absorption, therefore limited bond breaking and no material removal. As already observed in the lower ablation threshold range (50%), even near 50%, patterns are less dense, noisy, and poorly defined. At > 60% power, laser fluence is well above the ablation threshold, resulting in over-ablation and geometry degradation, and increased thermal accumulation. The excessive material removal per pulse creates loss of sharp edges, unontrolled periodicity and a transition toward irregular, deep, or merged features with reduced geometrical fidelity. Even in femtosecond regimes, excessively high energy leads to local overheating, plasma shielding, re-deposition of debris.

Based on preliminary laser texturing, the operational window of power setting 50 to 60 % is further detailed in the manuscript. The 50–60% power setting window captures onset of ablation, progressive structure refinement and consistent hydrophobicity evolution.

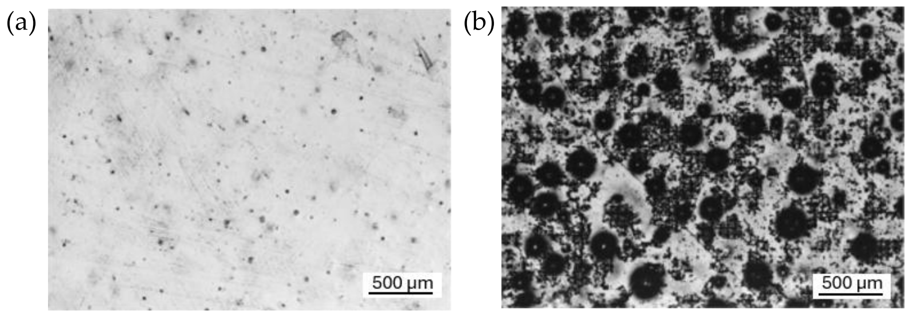

Figure S1. Microscopic picture of preliminary experiments when laser texturing outside the operational window of power setting, (a) < 50 % power setting, (b) > 60 % power setting.

 https://doi.org/10.3390/mi17060759

Supplementary information S2.

In addition to the selected data shown in Figure 4, the full data set for laser patterning of all coatings is shown in Figure S2.

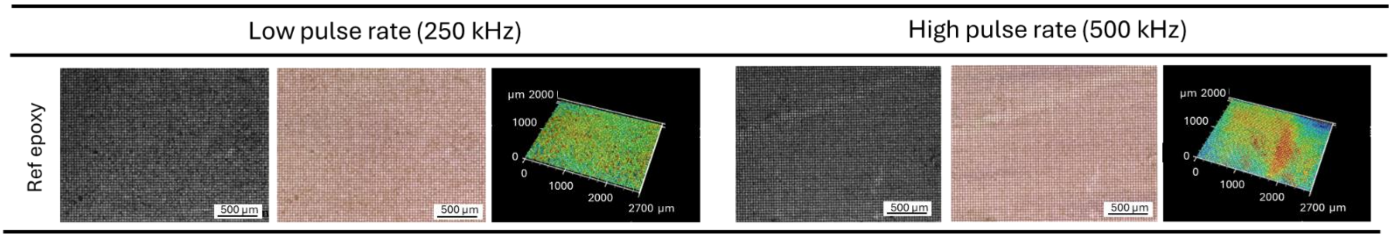

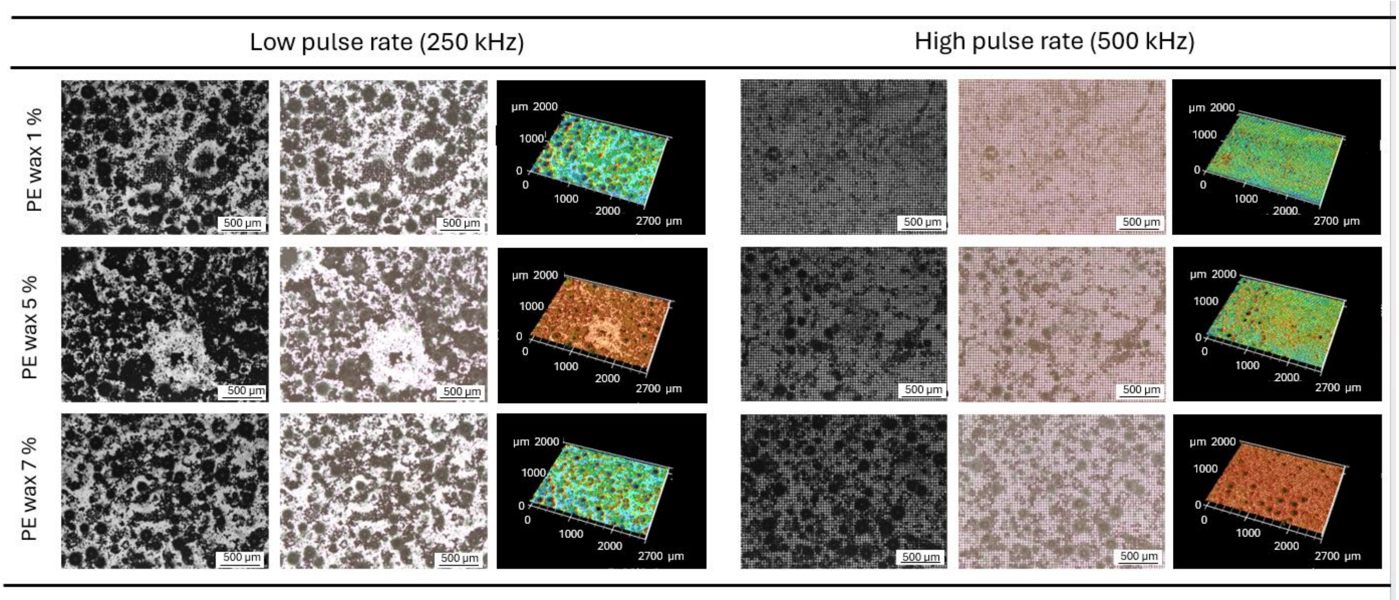

https://doi.org/10.3390/mi17060759

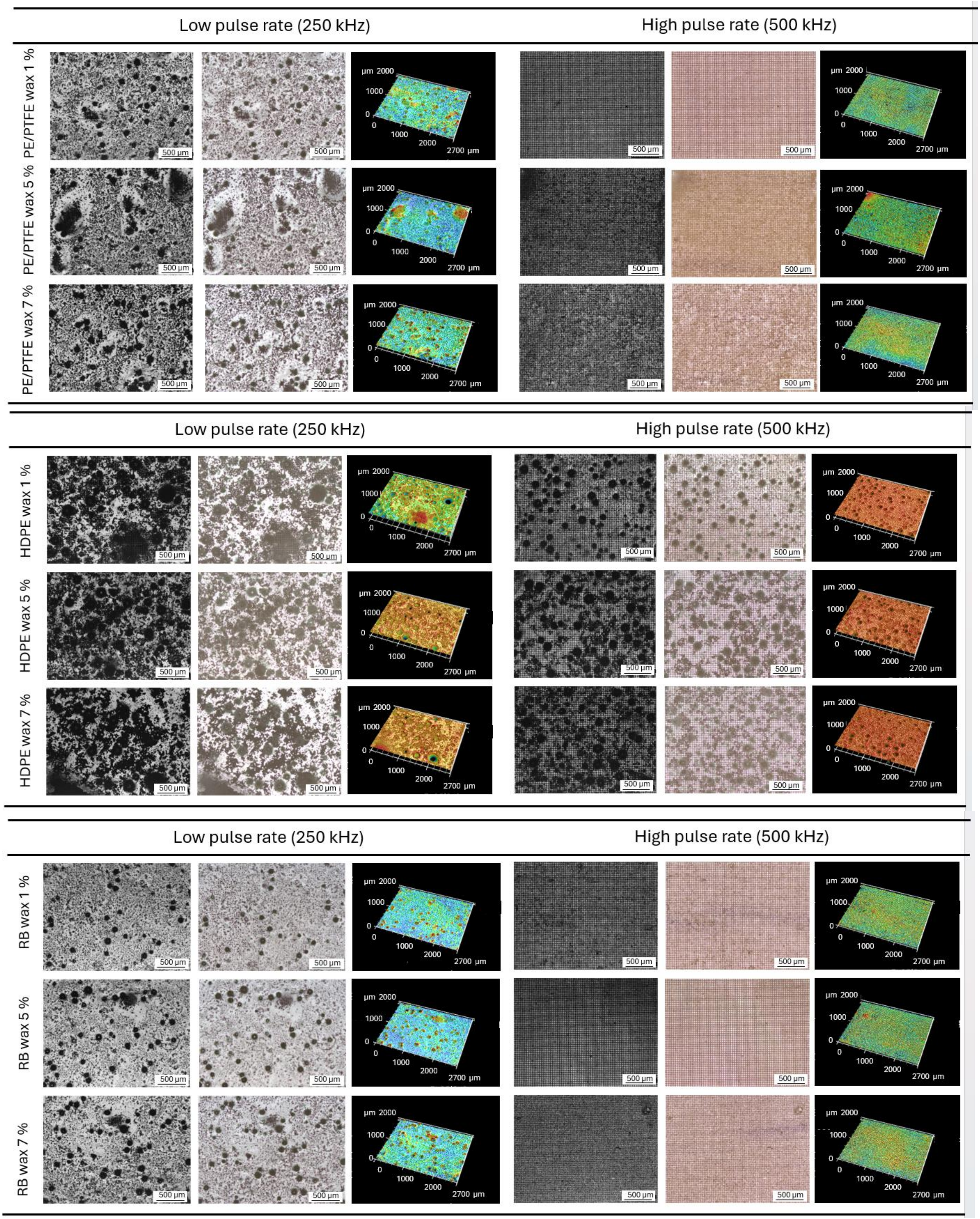

Figure S2. Full data set for long-range microscopic analysis of femtosecond laser patterned epoxy coatings with different types of wax fillers (fixed concentration of 7 wt.-%) processed under low pulse rate (250 kHz) and high pulse rate (500 kHz) and constant 60W laser power, including laser interference image, optical image and 3D topographical image.

https://doi.org/10.3390/mi17060759

Supplementary information S3.

Based on 3D topographical images recorded by laser interferometry, the average surface roughness Sa (µm) of the different coatings before and after femtosecond laser patterning is quantified in Figure S3.

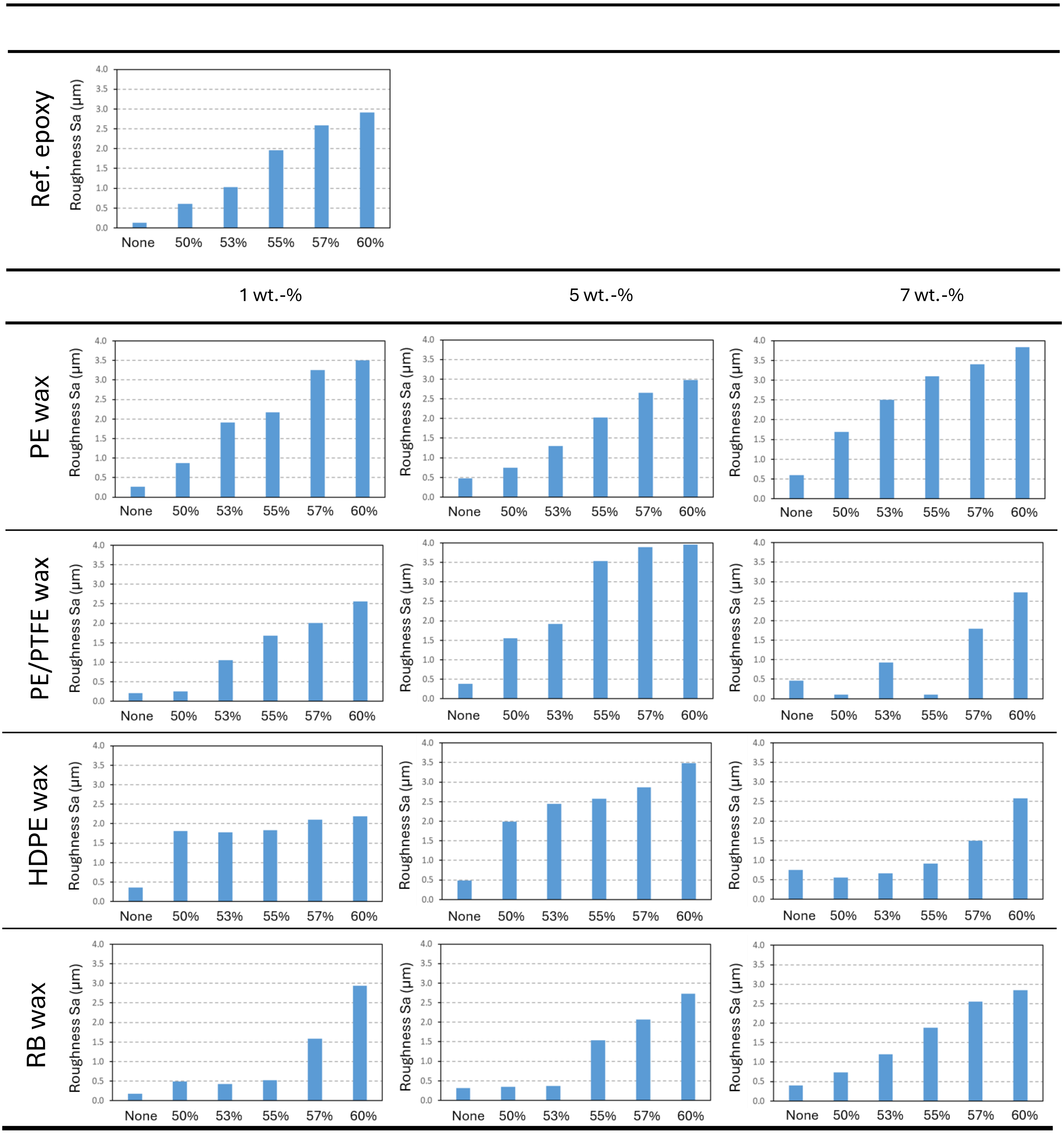

Figure S3. Average surface roughness Sa (µm) on reference epoxy coatings and wax-filled epoxy coatings with 1, 5, 7 wt.-% of PE wax, PE/PTFE wax, HDPE wax, RB wax, before and after femtosecond laser patterning at different laser power setting (50 to 60 %) (see X-scales, with “None” indicating the non-patterned coatings).

https://doi.org/10.3390/mi17060759

Supplementary information S4.

Determination of the ablation threshold value (power at given pulse rate 500 kHz) for laser texturing of different coatings. In Figure S4, red indicates unsuccessful development of a surface pattern, green indicates successful development of the surface patters.

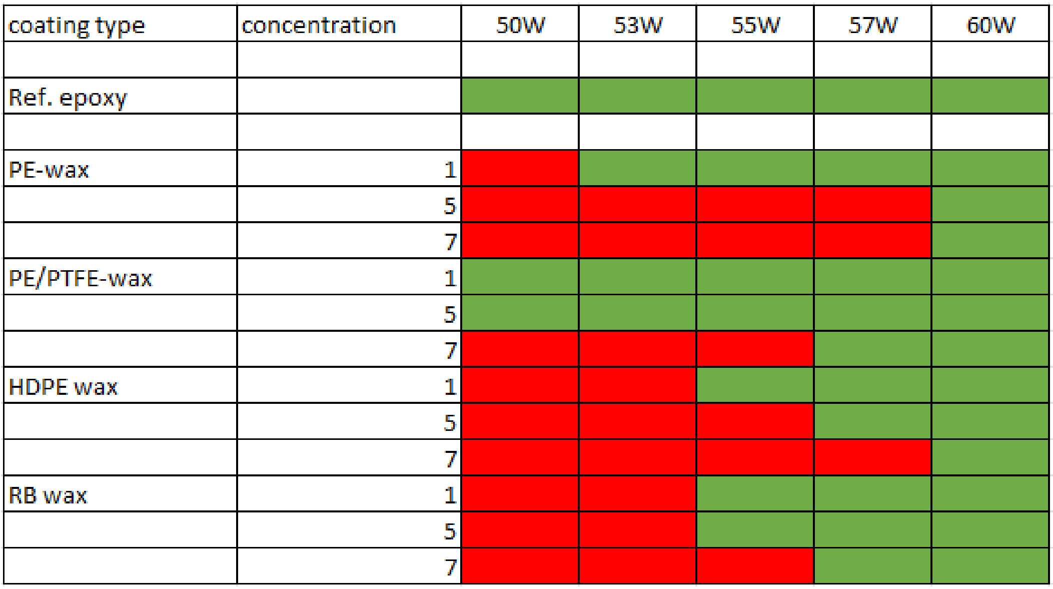

Figure S4. Determination of ablation threshold value for different coatings at 500 kHz.

Supplementary Information S5.

Contact angle measurements of the different coatings in initial state and after laser texturing at conditions of 60 % power setting, 500 kHz, 35 μm pitch distance are documented in Figure S5. The contact angle images are illustrated as an example measurement after laser texturing at maximum power setting, resulting in best improvement in hydrophobicity.

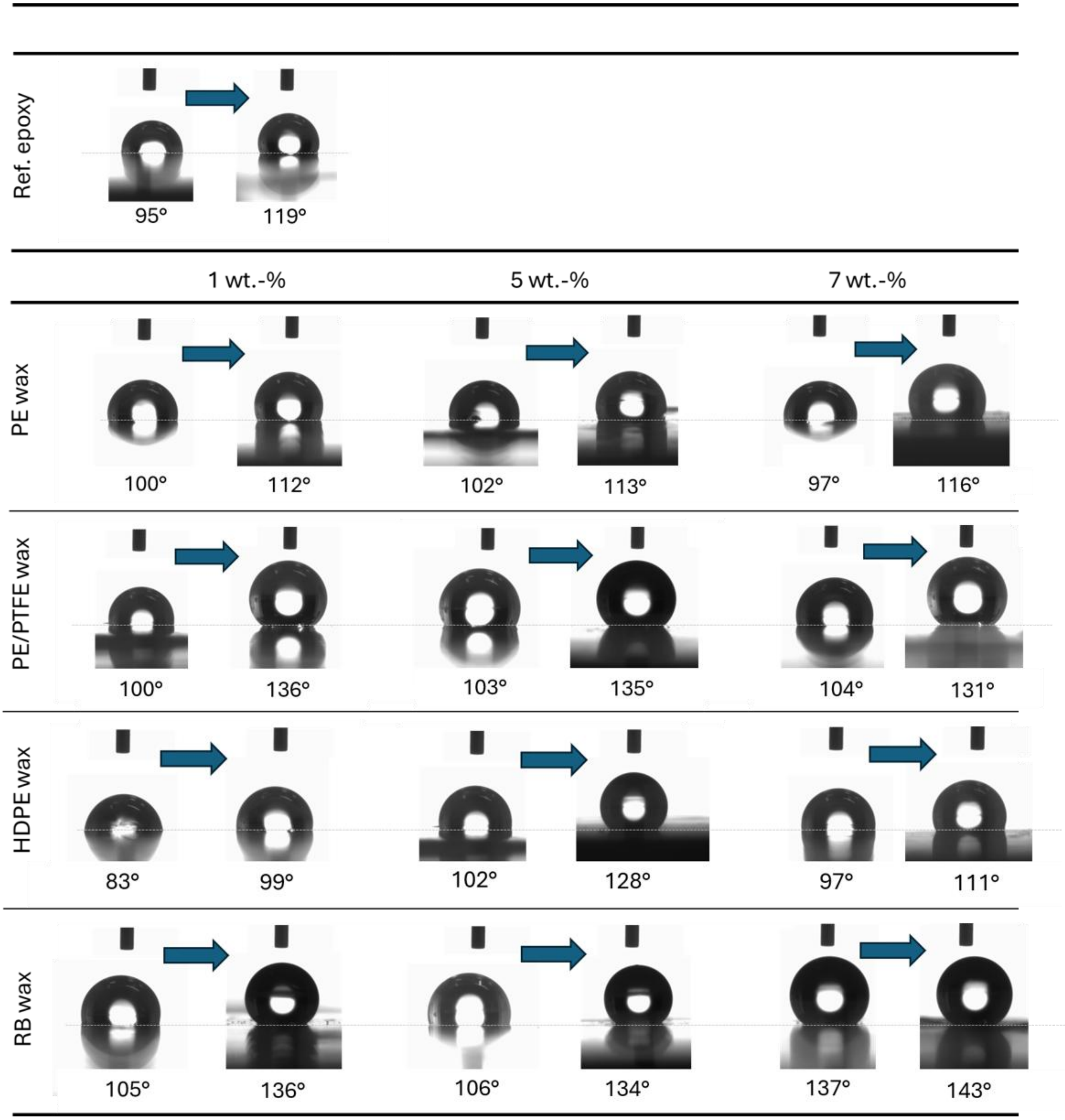

Figure S5. Illustration for static water contact angles on native coatings (before laser texturing) and after laser texturing at 60 % power setting, 500 kHz, 35 μm pitch distance, with indication of representative numerical value.

Supplementary information S6.

Based on 3D topographical images recorded by laser interferometry, the average surface roughness Sa (µm) of the different coatings before and after femtosecond laser patterning is quantified in Figure S6.

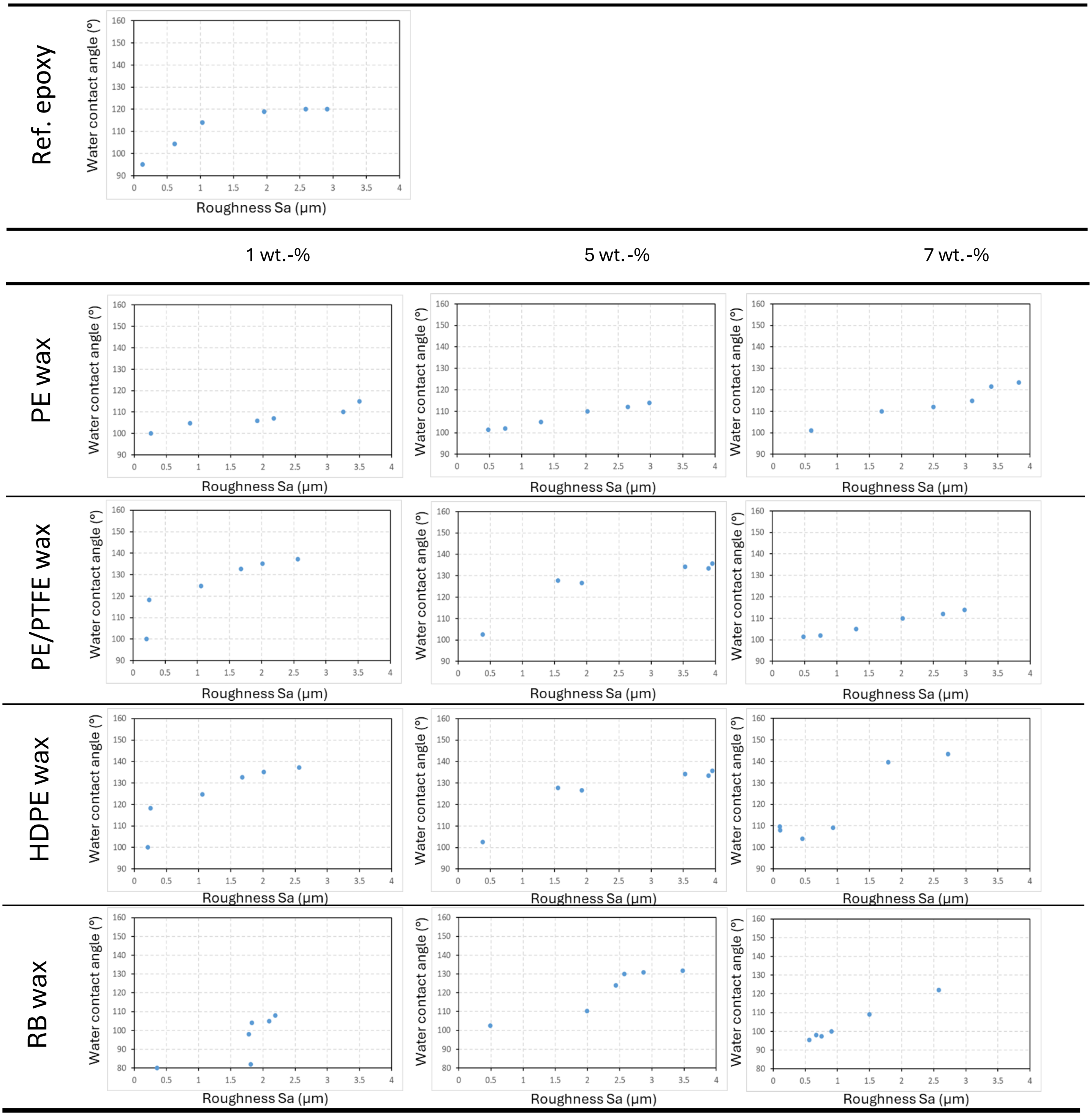

Figure S6. Direct relation between experimental measurements of water contact angles and average surface roughness Sa, including reference epoxy coatings and wax-filled epoxy coatings with 1, 5, 7 wt.-% of PE wax, PE/PTFE wax, HDPE wax, RB wax, before and after femtosecond laser patterning at different laser power setting (50 to 60 %).

https://doi.org/10.3390/mi17060759

Supplementary Information S7.

The variations in water contact angles are illustrated in Figure S7a under different conditions with an increase in water droplet volume and external vibration. The water droplets are deposited onto a selected coating that represents a maximum apparent contact angle of 143° (as reported in the text with a 3 μL water droplet), e.g., an epoxy coating with 7 wt.-% RB wax and femtosecond laser patterning at 60% power setting and 35 μm pitch distance. The changes in water contact angles depending on the droplet volume demonstrate a decrease in water contact angle with higher volume. It is known that the increase in pressure might cause a transition from the Cassie-Baxter wetting state (governed by pattern geometry and air entrapment) into the Wenzel state wetting (governed by roughness), thereby reducing the contact angle due to lack of effects of air entrapment. Alternatively, also the external vibration causes a loss of the Cassie-Baxter wetting state due to penetration of the water into the air pockets and wetting of the full substrate. As it is known that the Cassie-Baxter wetting state is a metastable condition, the results also confirm that the selected conditions with a water droplet of 3 μL is within the applicable range (2 to 5 μL droplets result in Cassie-Baxter wetting state).

A detailed microscopic image of the interface between the water droplet and a textured epoxy coating with 7 wt.-% RB wax is illustrated in Figure 7b.

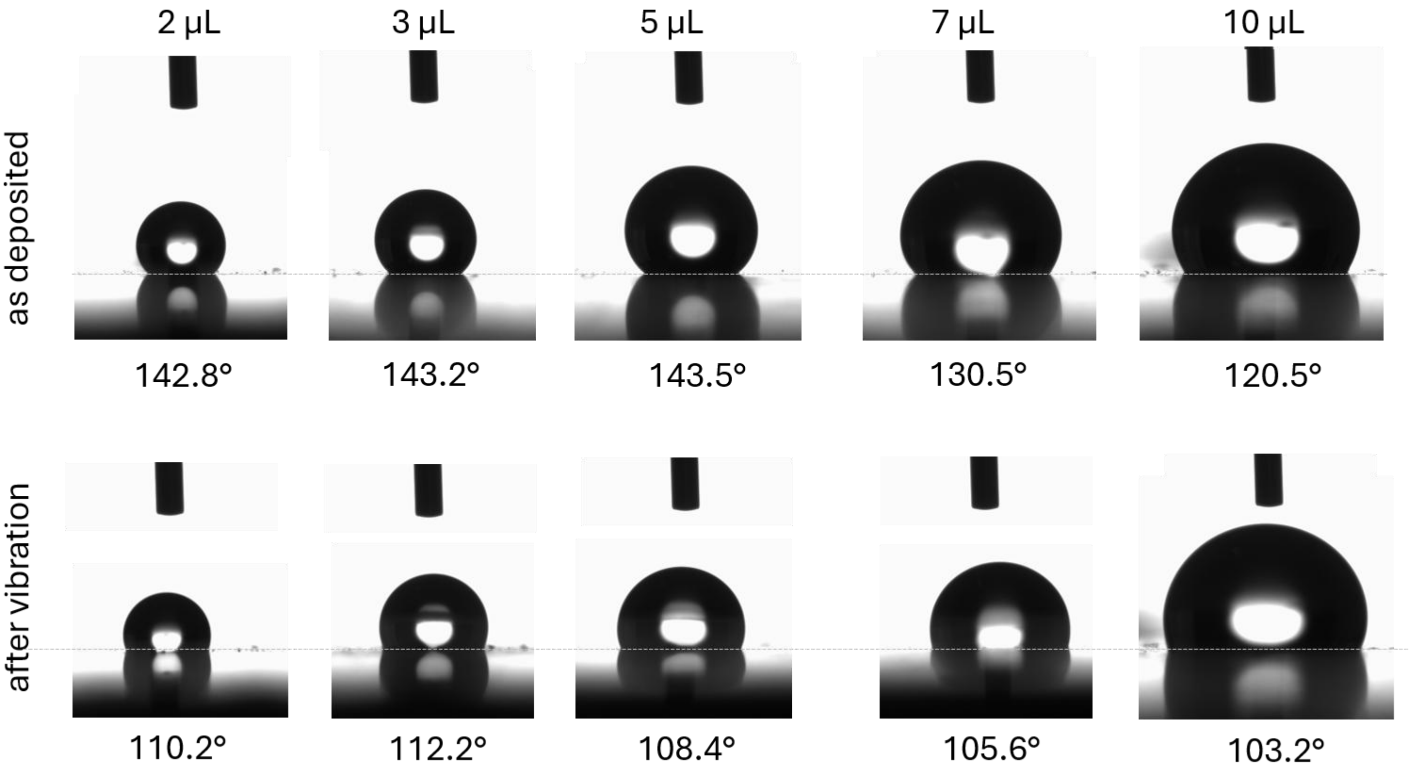

Figure S7a. Variations of water contact angles onto a laser pattered epoxy coating with 7 wt.-% RB wax depending on the water droplet volume and external vibration, with a transition from Cassie-Baxter wetting into Wenzel wetting at the higher droplet volume and with the application of external vibration.

https://doi.org/10.3390/mi17060759

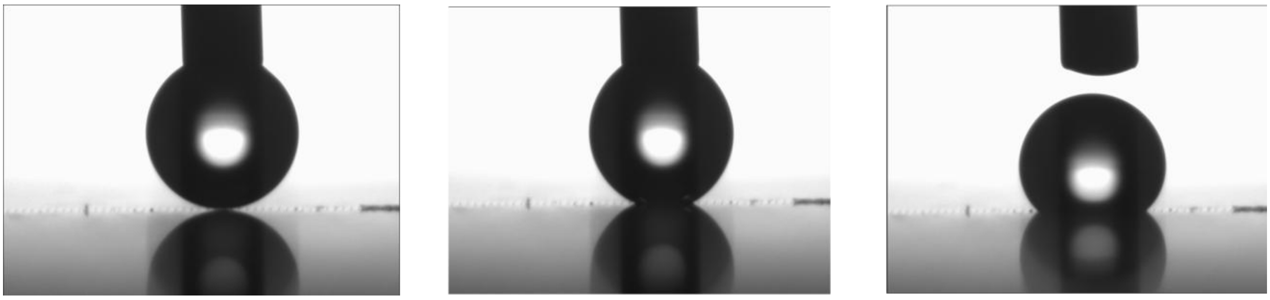

Figure S7b. Time-lapse frames for the deposition of a water droplet (1 μL) on a laser textures epoxy coating with 7 wt.-% RB wax, with a microscopic detail of the textured surface morphology.

https://doi.org/10.3390/mi17060759
